# Breast Cancer Risk, Fungicide Exposure and CYP1A1*2A Gene-Environment Interactions in a Province-Wide Case Control Study in Prince Edward Island, Canada

**DOI:** 10.3390/ijerph9051846

**Published:** 2012-05-11

**Authors:** Jillian Ashley-Martin, John VanLeeuwen, Alastair Cribb, Pantelis Andreou, Judith Read Guernsey

**Affiliations:** 1 Department of Community Health & Epidemiology, Faculty of Medicine, Dalhousie University, 5790 University Ave., Halifax, NS B3H1V7, Canada; Email: jillian.ashley-martin@dal.ca (J.A.-M.); pantelis.andreou@dal.ca (P.A.); 2 Atlantic Veterinary College, University of Prince Edward Island, 550 University Ave., Charlottetown, PE C1A4P3, Canada; Email: jvanleeuwen@upei.ca; 3 Faculty of Veterinary Medicine, University of Calgary, 2D02 TRW Building, 3280 Hospital Dr., Calgary, AB T2N4Z6, Canada; Email: acribb@ucalgary.ca

**Keywords:** breast cancer, pesticides, fungicides, cytochrome p-450, estrogen metabolism, epidemiology, gene-environment interactions, GIS-based exposure

## Abstract

Scientific certainty regarding environmental toxin-related etiologies of breast cancer, particularly among women with genetic polymorphisms in estrogen metabolizing enzymes, is lacking. Fungicides have been recognized for their carcinogenic potential, yet there is a paucity of epidemiological studies examining the health risks of these agents. The association between agricultural fungicide exposure and breast cancer risk was examined in a secondary analysis of a province-wide breast cancer case-control study in Prince Edward Island (PEI) Canada. Specific objectives were: (1) to derive and examine the level of association between estimated fungicide exposures, and breast cancer risk among women in PEI; and (2) to assess the potential for gene-environment interactions between fungicide exposure and a *CYP1A1* polymorphism in cases versus controls. After 1:3 matching of 207 cases to 621 controls by age, family history of breast cancer and menopausal status, fungicide exposure was not significantly associated with an increased risk of breast cancer (OR = 0.74; 95% CI: 0.46–1.17). Moreover, no statistically significant interactions between fungicide exposure and CYP1A1*2A were observed. Gene-environment interactions were identified. Though interpretations of findings are challenged by uncertainty of exposure assignment and small sample sizes, this study does provide grounds for further research.

## 1. Introduction

Breast cancer is the most common female cancer in North America, excluding non-melanoma skin cancer [1,2]. Breast cancer risk is not fully explained by known genetic inheritance, menstrual and reproductive history, or other established risk factors. The identification of possible modifiable breast cancer risk factors, therefore, is of considerable interest, including those that interact with other known breast cancer risk factors [3].

Numerous epidemiological studies have assessed associations between pesticide exposures and breast cancer incidence [4,5,6]. The majority of these studies have either described pesticide exposure generically or have focused on organochlorine and organophosphate pesticides. There is a paucity of studies of fungicide exposure and breast cancer despite evidence that fungicides have been toxicologically classified as carcinogens and endocrine disruptors [7,8]. One cohort study reported a statistically significant relationship between breast cancer risk and fungicides among women who had never used pesticides, but whose husbands reported exposure to captan in farming applications in Iowa (RR = 3.2 95% CI: 1.8–5.6). Statistically significant associations between breast cancer risk and exposure to maneb or chlorothalonil, however, were not observed [9]. Though several studies have examined the role of putative environmental risk factors (PCBs, tobacco smoke) in gene-environment interactions and breast cancer, no identified gene-environment interaction study has assessed fungicide exposure [10,11,12].The relationship between *CYP1A1* polymorphisms and breast cancer risk has been extensively studied. Several recent meta-analyses have reported that there is no over-all association with breast cancer risk for most of the common *CYP1A1* polymorphisms. Associations in subpopulations or with specific environmental exposures, however, may still be important [13,14,15,16]. Associations between *CYP1A1* polymorphisms and expression may occur through either changes in metabolism of estrogen or through changes in the metabolism of the environmental toxin. With regards to estrogen metabolism, *CYP1A1* has been predominantly identified as being involved in the formation of the 2-hydroxyestrogens, which exhibit lower estrogenic activity than the 4-hydroxyestrogens and ****16-alpha-hydroxy estrogens, metabolites that are believed to increase cancer risk [17]. Similarly, if metabolism of environmental toxins occurs at a higher rate in the presence of CYP polymorphisms, an individual’s risk of breast cancer may be elevated [18,19,20].

In this study, we chose to explore the possible interaction between a polymorphism in *CYP1A1,* a cytochrome p450 enzyme that is involved in estrogen and toxin metabolism [17], and fungicide exposure. The *CYP1A1* subfamily has been shown to interact with many environmental contaminants, most commonly through either induction in response to exposure or through involvement in metabolism [21,22]. Due to the potential endocrine disrupting and carcinogenic potential of specific fungicides, it is possible that this class of pesticides may impact breast cancer risk through either estrogenic or non-estrogenic pathways [7,8].

The present investigation is a further analysis of a recent case-control study that examined estrogen-metabolizing enzyme polymorphisms in relation to breast cancer incidence among women in Prince Edward Island (PEI) [23]. PEI provides a valuable study setting for this analysis as it is a rural province with a considerable percentage of land devoted to potato farming. The frequent use of fungicides on the potato fields and potential for atmospheric drift create potential for direct and residential exposure to these chemicals [24].

## 2. Methods

Specific objectives of this investigation were: (1) to assess the extent of association between breast cancer risk among PEI women and exposure to fungicides, derived using agricultural census data, land use files and geographic information systems (GIS) technology; (2) to explore the possibility of gene-environment interactions between breast cancer risk, *CYP1A1* enzyme polymorphisms and fungicides. The study design and participant recruitment were approved by the Human Ethics Committee of the University of Prince Edward Island and the Ethics Committee of the Queen Elizabeth Hospital, Charlottetown, PEI, Canada.

### 2.1. Study Population

The study population was the same population described by Cribb *et al.* [23]. Incident breast cancer patients (cases) (n = 207) were recruited from recently diagnosed breast cancer patients referred to the Queen Elizabeth Hospital (QEH) Medical Oncology Unit, in Charlottetown between July 1999 and March 2002 and referent participants (controls) (n = 621) were recruited from women presenting for routine mammography screening through the QEH Mammography Screening Program during the same period. As noted in Cribb *et al.* [23], 1,216 controls were recruited in the original case-control study, one withdrew from the study prior to analysis and sufficient DNA for analysis was not obtained from 12 recruits. Thirty-nine controls were identified as having breast cancer at the time of screening and, therefore, became cases. Of the 179 initial cases recruited from the oncology unit, seven were excluded due to inability to find appropriate matches and four were excluded because of insufficient DNA samples. Thus, 207 cases (168 initial cases plus 39 initial controls) and 621 controls were included in the final analysis. All study participants completed the study questionnaire during their clinic visit, thus non-response bias is not a concern. Women who had no previous personal history of breast cancer or abnormal mammography and were full time residents of PEI at the time of screening were eligible as controls. Controls were frequency matched 3:1 with cases on age (±5 years), menopausal status (current yes/no), and family history of breast cancer (mother, sister or daughter with breast cancer (current yes/no)). As the primary objective of the original case-control study was to assess the influence of single nucleotide genetic polymorphisms on risk of breast cancer, cases and controls were matched on age, family history of breast cancer, and menopausal status to facilitate analysis of the more subtle genetic influences on breast cancer risk [23].

### 2.2. Exposure Assessment

Fungicide exposure levels were derived using 1991 Canadian Agricultural Census data and census boundary files. The Agricultural Census provides data on the number of hectares in each consolidated census subdivision (CCS) that were treated with any fungicides. With these data, a continuous spatial surface of exposure, the percentage of land per CCS to which fungicides were applied, was developed using geographic information system (GIS) based methods [25]. The mean number of hectares per CCS (n = 64) was 6,930 (standard deviation 2,323) with a range of 3,557–12,782. Although there was some heterogeneity in the size of CCSs, this geographic unit of analysis was felt to have utility as a regional indicator of exposure.

Specific data on the type of fungicide used was not available from the census. All Canadian farm operators that produce agricultural products intended for sale, regardless of income, are asked to complete the Canadian Census of Agriculture. This census year was chosen to represent a 10 year estimated latency period between fungicide exposure and breast cancer diagnosis [26]. Moreover, as the census is only conducted every 10 years, it was determined that 1991 represented the most feasible and appropriate time period.

Participants provided their postal code, not residential address, in the original case-control study questionnaire. Thus, postal code, as a surrogate for residence, was mapped to the corresponding CCS using GIS, giving a percentage of land to which fungicide was applied for each participant’s CCS.

To allow for evaluation of multiple perspectives on the exposure disease relationship, fungicide exposure was analyzed as a continuous, binary, and categorical variable. The continuous exposure variable (percentage of land to which fungicide was applied) for each participant’s CCS was transformed into binary and categorical variables by assessing the frequency pattern of exposure among controls for notable changes in slope or breaks in the curve with cutoff assigned to minimize exposure misclassification. Using this technique, the binary variable was developed by assigning CCS with 10% or less of land treated with fungicides as low exposure and greater than 10% as high exposure. The histogram of exposure demonstrated a cluster of study participants below 10% and a scattered distribution of participants above 10%. Moreover, the selection of 10% as the cut-off point identified 14% of cases and 18% of controls as having high exposure. Selecting a higher cut-off point would have resulted in subgroups that were too small for analysis and selecting a lower cut-off point might not have isolated those individuals with high exposure. The categorical variables determined high exposure as greater than 10%, medium exposure as greater than or equal to 4.5% but less than or equal to 10%, and low exposure was determined to be less than 4.5%. These cutoff points were similarly determined by assessing the pattern of exposure distribution. As the objective of the original case-control study was to assess the influence of single nucleotide genetic polymorphisms on risk of breast cancer, there was no direct measurement of fungicide exposure during the initial study, nor structured collection of histories of past personal, residential or occupational exposures to fungicides as part of the questionnaire [23].

### 2.3. Genetic Polymorphism Data

Detailed methods for obtaining the genetic polymorphism data can be found in the original paper [23]. Buccal cells for DNA analysis were prepared using the protocol described by Richards *et al.* [27]. Genotyping of the CYP1A1*2A polymorphism (T3801C) was completed by PCR-RFLP according to the methods described by Bailey *et al.* [28] and then confirmed by the use of the National Cancer Institute Cancer Genome Anatomy Project validated Taqman^®^ assay for *CYP1A1* using primers and probes purchased from Applied Biosystems Inc (Assay number A-015943)**. **The CYP1A1*2A data were categorized into (1) homozygous referent and (2) heterozygous and homozygous variant alleles such that any influence of the variant allele was captured in the non-referent category. The heterozygous and homozygous variant alleles were grouped together in this manner to ensure sufficient sample size for comparison in the variant group.

### 2.4. Data Analysis

Descriptive statistics were calculated to compare effectiveness of matching variables (age, menopausal status, family history of breast cancer) and to assess breast cancer risk factor information. Crude odds ratios, t-tests and chi-square tests were examined to determine which variables were statistically significant associated with breast cancer risk. Covariates that did not change the effect estimate of fungicides on breast cancer risk by more than 10% were not included in the final logistic regression model. As cases and controls in this study were frequency matched, the analysis utilized unconditional logistic regression and included the matching variables [29]. Two models were developed.

Model I Analysis of the association between fungicide exposure and breast cancer risk adjusting for matching variables. Separate models were run for fungicide exposure as a binary, categorical and continuous variable.
Model II Analysis of the association between *CYP1A1* as a main effect and breast cancer risk, adjusting for matching variables. We explored the potential for a gene-environment interaction between the three fungicide exposure variables and the CYP1A1*2A allele by adding a multiplicative interaction term to the model and assessing the significance of the *p* value (Wald test) of the interaction term. All analyses were performed using SAS v 9.2 [30].

## 3. Results and Discussion

### 3.1. Descriptive Statistics

Matching of cases to controls according to age, family history of breast cancer, and menopausal status categories, proved to be effective; the distribution of these characteristics among cases and controls was nearly identical. Cases were significantly more likely to have a BMI greater than 25 at time of study enrollment, fewer children, and to have had an ovariectomy prior to age 45. Descriptive analysis also identified that cases were less likely (*p* value = 0.09) to ever use oral contraceptives (OC) (Table 1). As none of these four covariates changed the effect estimate of fungicide exposure on breast cancer risk by more than 10%, they were not included in the multivariate logistic regression models reported below.

**Table 1:** Univariate analysis of association between sociodemographic and lifestyle covariates and breast cancer risk, PEI, 1999–2003.

Variable	Level of Variable	Cases Mean (95% CI)	Controls Mean (95% CI)	Crude Odds Ratio	95% CI	*p* value ^1^
Age		54.6	55.0	0.99	0.98, 1.01	0.67
		(53.1–56.1)	(54.1–55.8)			
Duration of Residence on PEI (years)		44.1	42.7	1.00	0.99, 1.01	0.40
		(41.4–46.7)	(41.3–44.2)			
Age at Menarche (years)		12.9	13.0	0.96	0.86, 1.07	0.52
		(12.7–13.1)	(12.8–13.1)			
		N (%)	N (%)			
Positive Family History of Breast Cancer	Yes	53 (25.6)	159 (25.6)	1.0	0.70, 1.43	1.0
	No	154 (74.4)	462 (74.4)			
Menopausal status	Post	127 (61.4)	381 (61.4)	1.0	0.72, 1.38	1.0
	Pre	80 (38.7)	240 (38.7)			
Ethnicity	Caucasian	207 (100)	621 (100)	NA		
	Other	0	0			
Birthplace	PEI	150 (72.4)	472 (76.1)	0.82	0.57, 1.18	0.30
	Other	57 (27.5)	148 (23.9)			
Body Mass Index (at time of recruitment)	BMI ≤25	61 (30.2)	244 (39.80)	**1.53**	**1.09, 2.15**	0.01
	BMI >25	141 (69.8)	369 (60.20)			
Number of Children	None	31 (15.1)	64 (10.3)	1.0 ****	0.45, 1.17 ****	0.01
	1–3	137 (66.5)	389 (62.6)	0.73 ****		
	>4	38 (18.5)	168 (27.1)	**0.47**	**0.27, 0.81**	
Ovariectomy at <45 Years of Age	No	195 (94.2)	546 (88.2)	**0.46**	**0.24, 0.87**	0.01
	Yes	12 (5.8)	73 (11.8)			
Oral Contraceptive Use	Never	69 (33.5)	170 (27.4)	1.0 ****	**0.53, 1.05**	0.09
	Ever	137 (66.5)	451 (72.6)	**0.75**		
Currently Drink Alcohol	Yes	129 (62.9)	348 (56.7)	1.32	0.95, 1.83	0.09
	No	76 (37.1)	271 (43.8)			

^1^ *p* value from t-test for continuous variables and Chi-square test for categorical variables

### 3.2. Model I Association Between Breast Cancer Risk and Fungicide Exposure

After adjusting for matching variables in an unconditional multivariable logistic regression model, there was no statistically significant association between any of the fungicide exposure variables and breast cancer risk (Table 2). In both the binary and categorical variable, a higher percentage of controls (18%) than cases (14%) had high levels of exposure but a higher percentage of cases (16%) than controls (13%) had medium exposure in the categorical variable, though these finding were not significant.

**Table 2:** Unconditional multivariable logistic regression models ^a^ of association between breast cancer risk and fungicide exposure, PEI, 1999–2003.

Variable	Levelof Variable	Cases N (%)	Controls N (%)	Adjusted Odds Ratio	95% CI
**Fungicide Exposure** **(continuous)**	--	207	617	0.98	0.97–1.01
**Fungicide Exposure **	Low	178 (85.9)	507 (81.6)	1.0	0.46–1.12
	High	29 (14.1)	114 (18.4)	0.72	
**Fungicide Exposure**	Low	145 (70.0)	426 (68.6)	1.0	
	Medium	33 (15.9)	81 (13.04)	1.19	0.76–1.87
	High	29 (14.01)	114 (18.4)	0.74	0.47–1.17

^a ^Adjusted for matching variables (age, menopausal status, family history of breast cancer).

### 3.3. Model II. Association Between Breast Cancer Risk, Fungicide Exposure and CYP1A1*2A

The odds ratio between CYP1A1*2A and breast cancer risk was observed to be 0.78 (95% CI 0.55–1.13). Product terms between CYP1A1*2A and fungicide exposure were not significant in any of the exposure models (Table 3). The *p* value (Wald test) was smallest for the continuous exposure*CYP1A1*2A product term (0.14) and greatest for the categorical exposure*CYP1A1*2A product term (0.71) suggesting that the results were highly influenced by the low power in the exposure subgroups (Table 3).

**Table 3:** Unconditional multivariable logistic regression analysis of association between breast cancer risk, fungicide exposure and CYP1A1*2A allele, PEI, 1999–2003.

Variable	Level of Variable	Cases N (%)	Controls N (%)	Odds Ratio	95% CI	Product Term	*p* value
CYP1A1*2A ^a^	Referent	160 (78.4%)	465 (75.0)	0.78	0.55–1.13	*--*	--
	Variant	44 (21.6%)	154 (25.0)				
CYP1A1*2A ^b^	Referent	160 (78.4%)	465 (75.0)	0.78	0.55–1.13	CYP1A1*2Ax ****	0.14
	Variant	44 (21.6%)	154 (25.0)			**Continuous Exposure**	
CYP1A1*2A ^c^	Referent	160 (78.4%)	465 (75.0)	0.79	0.55–1.12	CYP1A1*2Ax ****	0.22
	Variant	44 (21.6%)	154 (25.0)			Binary Exposure	
CYP1A1*2A ^d^	Referent	160 (78.4%)	465 (75.0)	0.78	0.55–1.13	CYP1A1*2Ax ****	0.71
	Variant	44 (21.6%)	154 (25.0)			Categorical Exposure	

^a^ Adjusted for matching variables (age, menopausal status and family history of breast cancer); ^b^ Adjusted for matching variables and continuous fungicide exposure; ^c^ Adjusted for matching variables and binary fungicide exposure; ^d^ Adjusted for matching variables and categorical fungicide exposure.

### 3.4. Discussion

This further analysis of the PEI Breast Cancer Study was one of only a few case-control epidemiological investigations of fungicide exposure and breast cancer risk and the first to examine gene-environment interactions for genetic polymorphisms in p450 enzymes in relation to fungicide exposure. Though the present investigation did not identify a statistically significant association between fungicide exposure and breast cancer risk, toxicological evidence exists to support the role of fungicides as carcinogens and endocrine disruptors [7,8]. Chlorothalonil, one of the primary fungicides used on PEI, has been classified as a probable carcinogen by the U.S. EPA [7]. The primary metabolite of chlorothalonil (4-hydroxy-2,5,6-trichloroisophthalonitrile), known as DS-3701, has greater toxicity than its parent compound, as observed from wildlife studies [31]. The two other predominantly used fungicides on PEI, metiram and mancozeb, are both ethylene bisdithiocarbamates fungicides (EBDC) and have been classified as endocrine disruptors. Toxicological studies have observed that exposure to mancozeb induces apoptosis, thus providing a mechanistic link between mancozeb and cancer [32,33]. Moreover, the metabolite of EBDC fungicides, ethylene thiourea, has been classified as a carcinogen [34]. A cohort study that examined the association between maneb or chlorothalonil exposure and breast cancer risk did not identify an association between ever *vs.* never use of either chlorothalonil and breast cancer risk though this study was also limited by small samples sizes of exposed cases [9]. Metiram and mancozeb, the two other primary fungicides used in PEI were not assessed in the Iowa/North Carolina cohort study.

The present investigation’s observed that fungicide exposure was not statistically significantly associated with breast cancer risk. The trend in the models with binary and categorical exposure variables was towards a reduced risk of breast cancer risk among women with elevated levels of exposure. A biologically plausible explanation for this finding could be that fungicides inhibit the *CYP* p450 system and thus lessen the concentration of estrogen metabolites. Toxicological literature has reported that maneb, an EBDC fungicide with a chemical structure similar to mancozeb, inhibits *CYP* p450 enzymes, yet other supportive literature is lacking [35]. On the contrary, it is more likely that the potential carcinogenic effects of fungicides were not observed due to the limitations in sample size, bias, and misclassification. Further toxicological and epidemiological evidence is needed to clarify the relationship between fungicide exposure and adverse health outcomes.

The analysis of the interaction between fungicide exposure and CYP1A1*2A did not identify a significant product term for any of the exposure variables. As a main effect, presence of the heterozygous and homozygous variant CYP1A1*2A alleles suggested an inverse association with breast cancer risk, though was not statistically significantly. Biological interpretation of these observed findings is again limited due to the low sample size and potential influences of bias.

While no previous research reported in the literature has examined the interaction between fungicide exposure and the CYP1A1*2A allele in relation to breast cancer risk, studies of PCB exposure demonstrated that neither CYP1A1*2C status nor PCB exposure were independently associated with increased breast cancer risk [10,11]. However, women with the variant CYP1A1*2C allele who had the highest level of PCB exposure experienced an increased risk of breast cancer. Similarly, Li *et al.* [36] reported a trend among women with elevated levels of pesticide exposure towards increased breast cancer risk among subgroups of women with the CYP1A1*2C and CYP1A1*3 alleles.

In seeking an explanation for our findings, it is necessary to consider the dual role of *CYP1A1* activity. On the one hand, as *CYP1A1* is induced by many environmental contaminants, induction may increase the rate of estrogen metabolism toward the 2-hydroxyestrogens and away from the more estrogenic metabolites [12]. In this scenario, the interaction between the environmental exposure and the variant CYP1A1*2A allele could play a protective role because of an even greater activity of *CYP1A1*. On the other hand, *CYP1A1* has the potential to activate environmental toxins leading to an increased exposure to potentially toxic metabolites [37]. This latter role of *CYP1A1* is particularly relevant to the present investigation as the metabolites of both EBDC fungicides (ethylene thiourea) and chlorothalonil (DS-3701) are more toxic than the parent compounds. In this case, women with the variant allele may be at an increased risk of breast cancer due to an elevated concentration of these toxic metabolites. The possible relationships or interactions are therefore complex, and it is not possible to speculate from the current data on biological mechanisms or the significance of the putative association.

### 3.5. Strengths and Limitations

The primary strength of this investigation is the analysis of an understudied class of pesticides in a relatively high-exposure intensity geographical region. Moreover, the availability of detailed lifestyle and covariate data from the PEI case-control study allowed this investigation to evaluate potential confounding due to established breast cancer risk factors and the presence of genetic polymorphism data facilitated the analysis of gene-environment interactions.

In addition, PEI provides a valuable study setting due to the presence of a national healthcare system and relatively homogenous population. All residents of the island are provided with universal access to the national healthcare system, thus eliminating any financial or insurance barriers to screening or treatment. At the time of the study, the Queen Elizabeth Hospital was the primary oncology clinic and all breast cancer diagnoses on the island were recorded in the PEI cancer registry. During the study time period, 345 breast cancer diagnoses were recorded in the PEI cancer registry. The case-control study recruited 207 (60%) of these breast cancer cases, the geographic distribution of residential addresses of the case population was examined and shown to be representative of the overall pattern of disease on the island. In contrast to communities lacking a national healthcare system, women who reside in PEI may be more likely to seek routine mammography screening. Rates of mammography screening have been reported at 56% on the island [38]. The QEH mammography clinic is one of only two screening centers on the island. The QEH mammography clinic is located in the capital city, Charlottetown, and has two diagnostic imaging machines, whereas the screening clinic in Summerside is located in a community with lower population density and only one machine. It is estimated that two-thirds of PEI women undergo mammography screening at the QEH clinic. Moreover, more than 50% of the consolidated census subdivisions in PEI were represented by the study population (as indicated by a case or control residing in that subdivision). Thus, cases and controls provide a reasonable representation of the same underlying source population on the island.

The present investigation is, however, challenged by some exposure misclassification, and small sample size. While the use of the Agricultural Census data was a feasible means of assessing regional levels of fungicide exposure retrospectively, these data did not permit analysis of individual level exposure to specific fungicides. Moreover, reliance on postal code rather than individual residential address may have inappropriately assigned certain individuals to a CCS if they lived at the border of the CCS or collected their mail in a different location than where they resided, leading to errors in the percentage of hectares receiving fungicides assigned to some participants. These limitations highlight the challenges of utilizing a geographic based approach to exposure assessment. The inclusion of time-activity personal exposure histories and measurements of specific fungicides in environmental media would enhance precision of such analyses in future studies, but was not possible in this retrospective analysis of secondary data.

There are other considerations that must be taken into account in interpreting these results. PEI is relatively small, and given the extent to which fungicides are sprayed across the Island, the entire population may have in fact been exposed to elevated fungicide concentrations, as suggested by fungicide ambient air quality studies conducted by Environment Canada [39]. The inclusion of an external, off-Island control group would have strengthened the interpretation of study findings, but an external control group was not part of the original study. Moreover, while recruitment of the control population from the mammography clinic did minimize the potential for outcome misclassification among controls, this strategy does present potential selection bias as it may inadvertently exclude women who do not seek healthcare from the study population.

Lastly, the study was underpowered to detect associations between the estimated fungicide exposure and breast cancer risk, although the sample size was limited by the sample size of the original study. Utilizing the percent of exposure among cases and controls from the binary exposure variable, the study had 25% power to detect the observed associations, thus the likelihood of a Type 2 error is notably high. Moreover, the analysis of the interaction between CY1A1*2A polymorphisms and fungicide exposure also lacked sufficient power to make conclusive observations about the findings.

## 4. Conclusions

While there is limited data on relationships between breast cancer risk and exposure to insecticides and herbicides, there is a greater paucity of data for fungicides despite evidence of their toxic and endocrine disrupting properties. This investigation provided a valuable initial opportunity to explore such associations in relation to genetic predisposition and breast cancer risk in a region of documented intensive agricultural fungicide application. Fungicide exposure was not found to be significant associated with an increased risk of breast cancer, and no statistically significant gene-environment interactions were identified. Environmental fungicide metrics would have been enhanced by more precise exposure assessment methodologies. Further investigation into the health effects of this class of pesticides is warranted.
